# Illuminating Child Mortality: Discovering Why Children Die

**DOI:** 10.1093/cid/ciz562

**Published:** 2019-10-09

**Authors:** Pratima L Raghunathan, Shabir A Madhi, Robert F Breiman

**Affiliations:** 1 Center for Global Health, Centers for Disease Control and Prevention, Atlanta, Georgia; 2 Medical Research Council: Respiratory and Meningeal Pathogens Research Unit, University of the Witwatersrand, Faculty of Health Sciences Johannesburg, South Africa; 3 Department of Science and Technology/National Research Foundation: Vaccine Preventable Diseases, University of the Witwatersrand, Faculty of Health Sciences, Johannesburg, South Africa; 4 Emory Global Health Institute, Emory University, Atlanta, Georgia

**Keywords:** child mortality, surveillance, minimally invasive tissue sampling, verbal autopsy

## Abstract

Current understanding of the causes of under-5 childhood deaths in low- and middle-income countries relies heavily on country-level vital registration data and verbal autopsies. Reliable data on specific causes of deaths are crucial to target interventions more effectively and achieve rapid reductions in under-5 mortality. The Child Health and Mortality Prevention Surveillance (CHAMPS) network aims to systematically describe causes of child death and stillbirth in low- and middle-income countries using minimally invasive tissue sampling. The articles in this supplement introduce the set of foundational epidemiologic, demographic surveillance, social behavioral science, and laboratory methods. Undergirding the CHAMPS surveillance system designed to determine causes of child mortality.

## Teaser

Fundamental considerations behind the CHAMPS surveillance system are provided along with an introduction to articles included in this supplement.

Despite the progress made in reducing the annual global number of under-5 childhood deaths from 9.8 million in 2000 to 5.9 million in 2015, it is estimated that in 2030, 4.4 million children will still die before they reach the age of 5, assuming current downward trends continue [1]. The absence of data reliably characterizing the specific causes of death has severely hampered greater progress in preventing childhood mortality. Current understanding of the causes of under-5 childhood deaths in low- and middle-income countries is mainly shaped by country-level vital registration data and limited verbal autopsies. Although causes of death obtained from verbal autopsies and physician diagnosis are similar, both methods lack specificity in attributing pathogen-specific causes of death for many infection-related illnesses such as pneumonia, sepsis, and diarrhea [2]. Furthermore, verbal autopsies focus on the underlying cause of death without recognizing additional conditions in a causal chain, which, if successfully managed, could ultimately prevent the death. Studies on living children with severe illnesses, like the Global Enteric Multicenter Study (GEMS), have also been relied upon, often with the assumption that the causes of severe illness directly reflect causes of death [3]. Such guesswork with imperfect conclusions has been necessary because it is important to prioritize prevention of top killers, given limited public health resources.

One pathway through the abyss—studying postmortem specimens from children who have died, which can provide much more precise information—has, until recently, not been feasible, because of widely held beliefs that performing complete diagnostic autopsies on children would simply not be acceptable. Complete diagnostic autopsies (CDAs) are expensive, substantially delay burial time, and can present cultural barriers. While CDAs are generally not acceptable to parents, communities, and religious leaders, pioneering work has demonstrated that a quicker, less expensive, nondisfiguring, needle-based sampling approach for cause of death determination holds promise [4]. Minimally invasive tissue sampling (MITS, also known as minimally invasive autopsies) is acceptable, at least in some locations with high child mortality, and findings can be informative; studies in Mozambique have found reasonably high correlation of results from MITS and CDAs, especially for infectious diseases [5].

The Child Health and Mortality Prevention Surveillance (CHAMPS) network is being implemented at a time when heightened global focus on addressing massive inequities in childhood mortality has converged with the emergence of a variety of tools to characterize the specific infectious and noninfectious causes of death. The Bill & Melinda Gates Foundation has recognized that the time is right to generate mortality data in a way that is designed to enable stakeholders at local, national, regional, and global levels to prioritize the most effective strategies and invest in the most needed tools. Indeed, achieving the ambitious Sustainable Development Goal 3.2 (the elimination of all preventable under-5 mortality and stillbirths by 2030 and reducing global under-5 mortality to a maximum of 25 deaths per 1000 live births [from its current rate of 44 deaths per 1000 live births]), will require greater precision in public health and clinical approaches. Targeting the specific causes of child death will require more specific and robust data than have previously been available. Thus, the CHAMPS network represents a new, unparalleled effort to accelerate the pace of mortality reduction: CHAMPS seeks to systematically describe why children die in the parts of the world where children still die too often, and act upon the findings.

From the outset, we recognized the daunting challenges inherent in this charge. The surveillance system would have to detect and notify child deaths rapidly, as emerging data suggested that MITS would be more reliable in the first 24 hours after death. Yet the surveillance methods would have to be flexible enough for adaptation to vastly different country contexts in sub-Saharan Africa and South Asia. As a network, we would have to approach communities respectfully with a technical focus on child death, despite the fact that healthy and sick children may not routinely receive needed care. CHAMPS teams in country would have to gain acceptance from families, often including extended family members, to conduct postmortem investigations during a time of intense shock and grief. We would also have to implement standard procedures for MITS and pathology across countries with variable experience and clinical infrastructure. Other ambitious scientific goals included generating a set of laboratory diagnostic methods to diagnose a broad array of conditions relevant to pediatric mortality, and developing a standard way to analyze and integrate case-level information to assign cause of death. Finally, it was imperative to make cause of death and other actionable information (such as newly diagnosed human immunodeficiency virus, tuberculosis, and syphilis, among other infectious conditions) quickly available to families, host country public health systems, and the global public health community.

The aim of this supplement is to present a diverse compilation of methods that make this initiative so unusually compelling from a scientific standpoint. To illuminate all causes of mortality requires collaboration beyond a single discipline—hence the multidisciplinary nature of CHAMPS surveillance.

This supplement describes the set of foundational epidemiologic, sociobehavioral science, and laboratory methods behind the sophisticated public health surveillance system, employing MITS, that CHAMPS designed to probe child mortality through a uniquely configured partnership. Protocols and standard operating procedures were developed with scientific and technical expertise from Emory University, the Centers for Disease Control and Prevention (CDC), the Task Force for Global Health, Deloitte, the University of Barcelona, and from across the network of academic and nongovernmental organization collaborators in South Africa, Mozambique, Kenya, Mali, Bangladesh, Sierra Leone, and Ethiopia.

CHAMPS employs epidemiologic and demographic surveillance, as well as sociobehavioral science methods, to generate credible cause-specific mortality rates as surveillance data accrue over the coming years. In this supplement, Salzberg et al provide an overview of the design of the CHAMPS mortality surveillance system, which encompasses notifications of under-5 deaths and stillbirths from health facilities and communities, ideally within 24 hours, with the goal of complete ascertainment of eligible deaths from the catchment population. Argeseanu et al describe the existing and planned demographic surveillance systems that monitor CHAMPS catchment populations, which help with the crucial task of defining denominators for future cause-specific mortality rate calculations. Sociobehavioral science methods were critical to define how to adapt mortality surveillance to local community contexts, and many sites had to strengthen their qualitative research and community engagement capabilities prior to the launch of death reporting. The articles by Blevins et al and O’Mara et al present initial findings from the participant inquiry–based approach to community engagement and formative research conceptual frameworks, and demonstrate the success of the cross-network sociobehavioral science capacity building.

Innovative laboratory methods and newly developed pathology and molecular biology tools applied by CHAMPS are especially noteworthy and potentially applicable to other endeavors. Given the pivotal role of MITS in generating specific causes of death, we have included 2 articles on pathology. Ordi et al have developed MITS specimen collection techniques, standard MITS kits, training materials, and methods that were essential to launch the standardized specimen collection, pathology, and laboratory activities across the network and to achieve robust results in a multicenter study. Martines et al present the approach to histopathologic diagnosis and evaluation of postmortem tissue specimens that are routinely sent to the Central Pathology Laboratory at CDC. They have developed a telepathology network through which pathologists at multiple diverse CHAMPS surveillance locations review and discuss pathologic findings in partnership with the pathologists at CDC. Diaz et al describe painstakingly designed multiplex TaqMan Array Cards (TACs) for the efficient, locally processed molecular detection of 131 viral, bacterial, parasitic, and fungal pathogens of consequence in child and perinatal mortality. Diaz et al have trained the CHAMPS sites to implement TACs, and they continue to conduct quality assurance.

Blau et al have elaborated procedures and detailed diagnosis standards for the determination of cause of death. This “DeCoDe” review process synthesizes laboratory investigations and clinical and verbal autopsy data and enables local expert panels to assign causes of pediatric deaths and stillbirths and produce etiologies in a systematic, consistent way across the CHAMPS network.

Before CHAMPS surveillance began, one of us (S. A. M.) piloted a multidisciplinary investigation into child deaths as a proof of principle in Soweto, South Africa, ultimately a site in the CHAMPS network. The experience was revelatory on numerous fronts. Not only did families consent to MITS at higher rates (75%) than anticipated, but nearly all parents expressed relief and gratitude for being provided information about why their child died (see Madhi, Pathirani, Baillie, et al, in this supplement). In some cases, such as with the detection of perinatal death due to group B streptococcal infection, the information was used to counsel the mother regarding care to prevent adverse outcomes in future pregnancies. The process of bringing clinical and public health experts together to review child deaths highlighted gaps in care and brought new energy and advocacy for clinical and programmatic improvements within the hospital. The manuscripts describing the pilot findings from stillbirths, neonates, and children are presented as the capstone of this supplement (Madhi et al [6], Madhi et al [7], and Chawana et al [8]; in this supplement).

The current vision is that CHAMPS surveillance will be sustained for several years [9]. We believe that with time, as longitudinal information accumulates, CHAMPS will yield new insights into pathogens, structural conditions, and areas for improvement of clinical practice, health systems, and public health programs. Local analyses, data use, and intentional processes for evidence to action are built into the CHAMPS framework. CHAMPS data will be made available in real time, to maximize their global value and use for research, mortality prevention strategy development, advocacy, and training. Ultimately, this initiative seeks to shine spotlights to illuminate child mortality and to suggest and inform strategies that will make child deaths rare. We are heartened that from its inception, CHAMPS has fostered extraordinary cross-disciplinary collaboration among investigators, public health officials, civic and religious leaders and community members, and stakeholders in the South and North toward this aim.

The poet and novelist Adriano Bulla said, “The journey to the light starts with a candle. Once it is lit, darkness has gone forever.” While the greatest value of CHAMPS data will occur as the numbers accrue, we are already observing that the findings may shift paradigms on how to prevent child mortality. Specific data about factors contributing to childhood mortality emanating from CHAMPS are now rapidly available to a wide array of stakeholders (www.champshealth.org). We are partnering with many, including ministries of health, national public health institutes, the World Health Organization, the United Nation’s Children’s Fund, the World Bank, and others. Key questions remain: Will public health practitioners, clinicians, policy makers, pharmaceutical developers, researchers, donors, and local communities, among many others, be prepared to use the data and findings? And if necessary, will we pivot together and change course to take new effective actions to reduce child mortality?

## Notes

***Acknowledgments.*** The authors acknowledge the contributions of investigators who have developed the Child Health and Mortality Prevention Surveillance (CHAMPS) Network. They express gratitude to the families who have participated in CHAMPS.

***Financial support.*** This work was supported by the Bill & Melinda Gates Foundation (OPP1126780).

***Supplement sponsorship.*** This supplement is sponsored by the Emory Global Health Institute and the Bill & Melinda Gates Foundation.

***Potential conflicts of interest.*** S. A. M. has received grants from the Bill & Melinda Gates Foundation, Pfizer, Glaxo Smith Kline, Biovac, Novavax, and Sanofi Pasteur; and honoraria from The Bill & Melinda Gates Foundation. All other authors report no potential conflicts of interest. All authors have submitted the ICMJE Form for Disclosure of Potential Conflicts of Interest. Conflicts that the editors consider relevant to the content of the manuscript have been disclosed.
